# The Bottom-Up Rise Strength Transfer in Elderly After Endurance and Resistance Training: The BURST

**DOI:** 10.3389/fphys.2018.01944

**Published:** 2019-01-14

**Authors:** Tiziana Pietrangelo, Danilo Bondi, Edyta Kinel, Vittore Verratti

**Affiliations:** ^1^Department of Neuroscience, Imaging e Clinical Sciences, Università degli Studi “G. d’Annunzio” Chieti – Pescara, Chieti, Italy; ^2^Department of Rehabilitation and Physiotherapy, Clinic of Rehabilitation, University of Medical Sciences, Poznań, Poland; ^3^Department of Psychological, Humanistic and Territorial Sciences, Università degli Studi “G. d’Annunzio” Chieti – Pescara, Chieti, Italy

**Keywords:** resistance, endurance, aging, handgrip strength, MVC, systemic effect, cross-talk

## Abstract

The phenomenon of strength gain is highly relevant for sarcopenia and clinical aspect linked to aging. Recent advancements drive the interest toward the exercise-related cross-talk between distant tissues. We demonstrated the cross-talk between lower and upper limbs, we named the Bottom-Up Rise Strength Transfer (BURST), mainly linked to endurance training. In our opinion, this effect can be mainly related to systemic factors, likely circulating myokines and extracellular vesicles (recently defined in terms of “exerkines” and “exersomes”) whit an eventual concomitant reduction of a sub-clinical chronic inflammation. The neuronal mechanisms, even if to our sight less likely involved in this adaptation, need to be deeply investigated. Further studies are needed to better characterize the exercise-related BURST, concerning the specificity of different protocols and the underlying physiological mechanisms.

## Introduction

The gain of strength is a very interesting physiological effect for the hypotrophic and dynapenic phenomena, in particular for the elderly, who experience year by year a decremental strength along with a diminishing muscle mass, a physiological condition named sarcopenia (Palmio and Udd, 2014). The field also comprises the intriguing topic of strength transfer: Rønnestad et al. (2011) reported a significant superior relative improvement in 1RM biceps curl following the combined leg and arms training (from 14.0 to 20.8%), compared to the exclusive arms training.

While this specific facilitation effect on strength transfer has been recently introduced, the phenomenon of cross-education (or cross-training or contralateral strength training effect) is known over more than a century (Scripture et al., 1894): it has been defined as a strength increase not only in trained but also in homologous untrained limbs after RT (Munn et al., 2004, 2005).

Even if several confounding factors could be invoked to limit the power of these phenomena, the physiological effect of cross-education is estimated to a contralateral strength gain of 11.9% (Manca et al., 2017).

Seminal studies revealed that several neuronal pathways from cortical to spinal level are involved in the cross-talk between homolateral and contralateral homologous trained muscle (Farthing et al., 2005; Carroll et al., 2006; Leung et al., 2018). The cross-education has been explained by two models of neuro-plasticity based on either “bilateral access” of motor engram by untrained circuit or on “cross-activation” of motor pathways (Lee and Carroll, 2007; Ruddy and Carson, 2013; Hendy et al., 2017).

It is worth mentioning that the published studies related to both cross-education and cross-transfer between lower and upper body were mainly conducted in young subjects (Hansen et al., 2001; Munn et al., 2004; Hendy and Lamon, 2017; Bartolomei et al., 2018). More recently, Ben Othman et al. (2018) discussed cross education phenomena also in developmental stage. Indeed, the elderly could take a big advantage from these phenomena, but to our knowledge, no study has been addressed to the cross-transfer between legs and arms in elderly.

In cross-education studies, as well as in the cross-transfer ones between lower and upper body, the conditioning protocols are related to RT. Even though several training protocols produce systemic effects (Sapp et al., 2016) and specifically the ET has sought to be highly related to systemic phenomena (Mikkelsen et al., 2013), the role of ET on cross-transfer has not been investigated yet.

Recently some research groups hypothesized biochemical factors to be involved in cross-education effect (Hendy and Lamon, 2017; Whitham et al., 2018): in this manner, systemic effects due to factors released by contraction might promote the functional adaptation of untrained muscles. In the same manner, these likely factors could also be considered in the cross-talk between distant tissues.

To best of our knowledge, in the cross-transfer studies has been addressed only the combination of upper and lower body exercises. We, therefore, hypothesized that cross-transfer of strength from lower to upper limbs could also be present in elderly following training dedicated exclusively to lower limbs. Furthermore, we hypothesized that the cross-transfer could be also related to ET. Thus, we designed a specific study on healthy elderly investigating the effect of both ET and RT performed specifically on their big muscle groups of lower limbs.

## Materials and Methods

### Participants

We recruited 31 healthy male (71.77 ± 4.06 years, 80.47 ± 12.09 kg, 1.67 ± 0.08 m), reporting current physical inactivity during the initial interview. Exclusion criteria were: irregular ECG, osteoarticular pathologies, mild to medium cardio-circulatory pathologies, diabetes type I or II, not-controlled hypertension, cancer, neurological or psychiatric disease, respiratory pathology, neuromuscular disease, genetic disease. All participants provided their written informed consent. The study was approved by the Ethics Committee of the Università degli Studi “G. d’Annunzio” Chieti–Pescara, Italy (protocol no. 1233/08 COET).

### Experimental Design

We used an experimental longitudinal design. The subjects were divided randomly into three groups: ET, RT, and control (Table 1). All the following tests were executed before (*t*0) and after (*t*1) the completion of the two different training protocols, detailed below. For the control group we respected the same ex-post time of other groups.

**Table 1:** Anthropometric characteristics of the subjects, before and after the relative protocol (Mean ± SD).

	**Control**		**Endurance training**		**Resistance training**	
	
Participants	10		10		11	
Age	70.80 ± 3.08		73.10 ± 4.93		71.45 ± 4.03	
	
	**Ex**	**Post**	**Ex**	**Post**	**Ex**	**Post**
	
BMI (kg/m^2^) FM (%) Inf. circ. (cm) Int. circ. (cm) Sup. circ. (cm)	29.02 ± 3.06 27.02 ± 4.84 38.79 ± 1.90 45.76 ± 2.08 53.21 ± 2.47	29.16 ± 2.93 27.02 ± 4.84 39.23 ± 2.05 46.53 ± 1.77 53.49 ± 2.47	28.31 ± 3.46 27.38 ± 4.25 40.85 ± 3.21 47.22 ± 2.89 54.70 ± 3.47	27.99 ± 3.16 27.31 ± 5.95 41.04 ± 3.15 48.25 ± 2.85 55.27 ± 3.77	29.91 ± 3.94 27.75 ± 5.36 41.53 ± 4.55 47.74 ± 5.24 55.21 ± 4.94	29.75 ± 3.74 25.94 ± 6.91 41.75 ± 4.46 47.16 ± 5.19 55.02 ± 5.58


*BMI: Body Mass Index; FM: Fat Mass; Inf. circ.: inferior circumference of dominant lower limb; Int. circ.: intermediate circumference; Sup. circ.: superior circumference; no significant difference was found in the following parameters.*

### Anthropometric Data

We measured the height and the weight of the subjects, and calculated their BMI. We estimated the percentage of FM using the plicometric method (Durnin and Womersley, 1974). We measured the circumferences of the dominant lower limb at three levels: inferior, intermediate, and superior (Benelli et al., 2007).

### Strength Assessment

To assess bilateral isometric strength of quadriceps, we used MVC method on a leg extension machine (Nessfit NMI 1000, Bcube, Italy) equipped with a dynamometer (Tesys 800, Globus, Italy). The subjects sat positioned with knee and hip joint angle at 90°, and they were required to carry out maximal isometric contraction for 5 s using both legs contemporarily. The test was carried out three times with a recovery time of 2 min and the best performance was considered. We focused on quadriceps to adhere with the training protocols.

Maximal HS was measured with a specific dynamometer (T.K.K. 5101 GRIP-D, Takei Scientific Instruments, Japan). The subjects remained standing up and performed their maximal grip strength with the dominant upper limb, with the arm straight at their side. The test was carried out three times with recovery time of 2 min, and the best performance was considered. We focused on HS to provide a different functional model respect to the lower limb tests and to provide a widely used, simple and highly reliable test.

### Protocols

Considering our group as older adults who were not active, we used a multiple-months progressive intervention, with a regular monitoring and a re-evaluation of the plans in accordance with functional changes (Nelson et al., 2007). Training consisted of three sessions per week, for a period of 12 week. Before each session, there was a warm-up period of 5 min on the cycloergometer at low intensity. After each session, there was a cool-down period of 5 min pedaling on the same cycloergometer at low intensity, followed by 5 min of stretching exercises for lower limbs.

Endurance Training was set in accordance to the minimum recommendation for vigorous-intensity aerobic activity in older adults of 20 min on three days each week (Nelson et al., 2007). The subjects were trained on a cycloergometer, pedaling at a constant intensity monitored through Heart Rate (HR) by an HR monitor (Polar Accurex Plus, Polar, Finland). HR training (HR_tr_) was calculated according to Tanaka formula (Tanaka et al., 2001) and Karvonen formula (Karvonen, 1996). Intensity was set at 0.6–0.7 (1st–4th week, 30’ pedaling; 5th–8th week: 40’ pedaling) or 0.8 (9th–12th week: 40’ pedaling) of HR_tr_.

Resistance Training was set coherently with studies of contralateral training effect (16 controlled studies, range 4–12 week, 15–48 training session) (Carroll et al., 2006). The subjects were trained on leg-press and leg-extension machines, setting the number of series (three, both on LP and LE machine) in accordance with the evidence for contralateral training effect (Munn et al., 2005). Our plan met the minimum recommendation of two days each week for muscular strength in older adults, involving major muscle groups (Nelson et al., 2007). The subjects were trained with three series on leg-press and leg-extension machines. Intensity was based on the 1RM: 1st–4th week, 12 repetitions at 60% RM; 5th–8th week, 10 repetitions at 70–75% RM; 9th–12th week, 6–eight repetitions at 80% RM.

### Statistical Analysis

The statistical analysis was carried out using GraphPad Prism Software, version 7 (GraphPad Software, La Jolla, CA, United States). To verify if the number of subjects was adequate to the present multiple comparisons, we used the following estimation:

$$n\;\geq \;2{\text{φ}}^{2}{\left(t(\text{α},\text{υ})\;+\;t(\text{β},\text{υ})\right)}^{2}$$

Setting φ = 0.5, α = 0.05, β = 0.1, and ν = 27, the result was *n* ≥ 5.66. Thus, we considered the minimum of six subjects for each group. Our results encountered this requirement. Normality of the distributions was assessed with D’Agostino and Pearson omnibus test. Data are reported as Mean (M) ± Standard Deviation (SD). Two-way ANOVA for Repeated Measures was used to analyze differences between groups and over the two time spots, in addition to Sidak (for initial and final differences between protocols), and Holm-Sidak (for ex-post significance of each protocol) *post hoc* analysis for multiple comparisons. We used correlation matrixes with Pearson or Spearman method to test the correlations between parameters at *t*0 and *t*1 and between the variations (calculated as ratio of *t*1/*t*0 values).

## Results

### Strength Assessment

**FIGURE 1:**
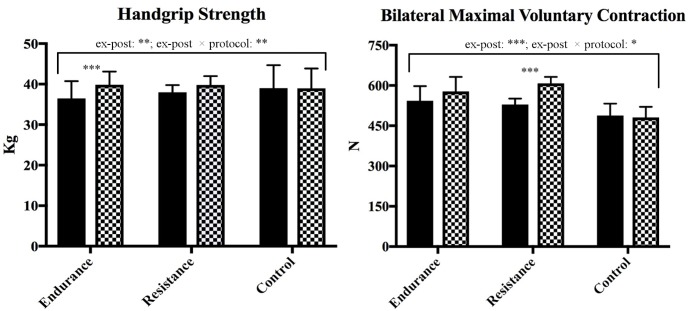
Results of HS and MVC, expressed as Mean ± SD. For each group (endurance, RT, and control), close columns represent the ex-post course (black columns represent *t*0 values, black and white columns represent *t*1 values). The *post hoc* analysis revealed the significance for endurance on HS and for resistance on MVC. ^∗^*p* < 0.05, ^∗∗^*p* < 0.01, ^∗∗∗^*p* < 0.001.

Regarding MVC on lower limbs, we found a significant increase from *t*0 to *t*1 (Figure 1), with a time effect (*p* < 0.01) and an interaction effect (*p* < 0.01); *post hoc* analyses revealed a specific positive effect (*p* < 0.001) only in the resistance protocol.

Regarding HS, we found a significant increase from *t*0 to *t*1 (Figure 1), with a time effect (*p* < 0.001) and an interaction effect (*p* < 0.05). *Post hoc* analyses revealed a significant strength increase in the upper limbs (*p* < 0.001) in the endurance protocol, and a similar tendency in the resistance protocol (*p* = 0.061). In all the comparisons, Sidak *post hoc* test didn’t reveal any statistical difference at *t*0. Correlation analyses highlighted a positive relation (*r* = 0.427) between HS and MVC at *t*0, not related to a specific protocol.

### Anthropometric Characteristics

From *t*0 to *t*1, there were no significant differences in BMI, in the percentage of FM and the three circumferences of dominant thigh (Table 1); there was a tendency toward an increase in inferior circumference by time effect (*p* = 0.062). In all the comparisons, Sidak *post hoc* test didn’t reveal any statistical difference at *t*0. Correlation analyses highlighted a positive relation (*r* = 0.447) between the variations of FM and Superior Circumference, not related to a specific protocol.

## Discussion

Dynamic, static, and isokinetic muscle strength physiological decreases with age. The association of age-related loss of muscle mass and functional decline invalidate people in their independent mobility as such as in Activities of Daily Living. The physical exercises, strictly linked to the principle of “Use it or lose it” (Rea, 2017), are among the most important stimulators of human muscle physiology. The simple design we applied to our study, ET and RT exclusively on lower limbs, permitted us to clearly observe the independent effect of lower limb training on upper limb strength gain, a specific strength cross-transfer we named the BURST. To our knowledge, this paper represents the first description in the literature of the BURST effect on elderly people. In addition, the idea that the skeletal muscle contraction produces positive effect not only on the contracting muscle itself, but also to other distant skeletal muscles no actively recruited to contraction, enriches the paradigm of exercise as powerful human physiology stimulator.

Our results demonstrated that both training protocols increased MVC on lower limbs, and, as expected, the RT showed more effectiveness with *post hoc* significance (Onambele-Pearson and Pearson, 2012; Burich et al., 2015).

More interesting, we observed that these training exerted a positive effect on untrained upper limbs. Indeed, we can’t completely exclude the activation of upper limb muscles during cycling and resistance exercises. In fact, during cycling, the arm muscles hold the handlebar to maintain balance and permit a more efficient pedalling. While during leg extension and leg press tasks the handgrip permit to improve the leaning due to better biomechanical facilitation. In any case, in both conditions, the upper limbs were used only for proper biomechanical stabilization and never directly trained.

The specific effect of ET on HS and a similar tendency for RT, may lead the investigation toward three main mechanisms: the mediating neuronal processes, the release of biochemical factors and the oxInflammation modulation.

The demonstrated retention of cross-education effects drives the nervous interpretation toward the fundamental role of central adaptations, with the contribution of motor learning to cross-education (Green and Gabriel, 2018). Both common drive to both hemispheres based on the adaptations of motor centers and interhemispheric access of motor engrams (Lee and Carroll, 2007; Ruddy and Carson, 2013; Green and Gabriel, 2018) are therefore related to specific motor plans.

Considering the differences in the motor programs and the pathways related to upper and lower limbs performances included in our study, and considering the simple coordinative requirements of HS test, although we did not attempt to determine the neural mechanisms, we consider the nervous system to be less likely involved in BURST.

Considering the common course of strength adaptations on neuromuscular basis (better activation, optimized synchronization of motor units and de-recruitment of antagonist muscles) and the maintenance of neuroplasticity in elderly (Barss et al., 2016), further studies are nevertheless needed to clarify the prospective neural mechanisms underlying the BURST. These studies may address the acute and chronic potential mechanism and sites of cross-education argued recently by Frazer et al. (2018), including several cortical motor and non-motor regions and the entire neuroaxis.

Although we have just preliminary results, and we did not attempt in this perspective study to determine the biochemical factors likely involved on the BURST, several candidates can be invoked, such as epigenetic, redox adaptations and regenerative system (Pietrangelo et al., 2012, 2015). These systems should therefore be addressed in a combined framework, as highlighted by Crippa et al. (2012) about the exercise-mediated effect of several miRNA on the fine regulation of satellite cells. The specificity of different training protocols have been demonstrated for several circulating-miRNA (miR-21, miR-221, miR-222, miR-146, and miR-486), with time-dependent changes (Sapp et al., 2016). Horak et al. (2018) proposed to differentiate between responders and non-responders in terms of circulating-miRNA following different training protocols, highlighting a selectivity for miR-222 with RT and for miR-21 with ET on young men. The field of transcriptomics could be deeper clarified also investigating the circular RNAs, a new class of post-transcriptional regulators with high stability, resistant to the enzymatic degradation, which are promising biomarker for clinical research (Maass et al., 2017). Indeed, the biochemical regulators on blood flow could be also vehiculated by exosomes, which are secreted by cells and released in the extracellular *melieu* where they could keep in contact with the surrounding cells or could be captured into the flowing blood or the lymph and addressed to the entire body. It is worth mentioning that the ET more effectively activates the cardio-vascular apparatus with respect to RT.

The model of “exerkines” is promisingly supported by the discovery of apelin as a powerful peptide able to prevent sarcopenia (Vinel et al., 2018) as well as other myokines like irisin, IL-6 and IL-8 playing a beneficial role in cell adaptation in response to exercise (Díaz et al., 2018).

The model of “exerkines” and “exersomes” considers the importance of humoral factors, reversed into the circulation, to promote cross-talk between distant tissues and organs, improving the beneficial effects of physical exercise. This model may be a great candidate to explain the pro-metabolic effect of endurance exercise (Safdar et al., 2016). The Non-Local Muscle Fatigue effects may also be considered in the exercise-related systemic cross-talk: this model involves the interconnected physiological pathways, the high dependence on muscle groups, the gender differences and the lifelong training habitus (Halperin et al., 2015). In addition, we have to pay attention to the recruited muscle group. Some studies have targeted small muscles which are the least likely candidates to provide systemic effects (Hendy and Lamon, 2017).

Microvesicles/exosomes stuffed with miokines and hormonal factors (Hendy and Lamon, 2017; Whitham et al., 2018) might be involved in cross-talk, exerting similar improvements demonstrated in training-derived remote strength transfer as a consequence of blood flow restriction during resistance exercises (May et al., 2018). Our group demonstrated that adult skeletal muscle stem cells specifically stimulated could release exosomes stuffed with guanosine-based molecules (Pietrangelo et al., 2018).

Frühbeis et al. (2015) demonstrated the extra-cellular vesicles release after a single bout of endurance exercise, especially in the moderate-intensity phase and with a higher response of exosomes, providing a possible explanation of distal signaling during moderate-to-vigorous intensity exercise.

Controversial results exist about the hormonal factors: there is evidence for the mediating role on cross-transfer (Hansen et al., 2001) but also for the lack of the hormonal role in cross-education after a few weeks of training (Beyer et al., 2016). Thus, further studies on the field should address the role of several candidates, such as myostatin (Walker et al., 2004), GH and testosterone (Rønnestad et al., 2011) in middle-term systemic effects, differentiating the amount of mediation effect between different training protocols.

For a comprehensive interpretation of BURST, we should also consider the redox system and its implication in several adaptive response (Griffiths et al., 2014). Valacchi et al. (2018) recently proposed the term “OxInflammation” to define the sub-clinical pro-inflammatory response that can turn in a chronic physiological dysfunction and impairment. We demonstrated (unpublished preliminary data) that ET significantly decreases the presence of anion superoxide into the skeletal muscles with respect to the RT, improving the muscle oxidative status.

Moreover, we tested the Free Oxygen Radical Defense (Callegari) on peripheral blood and we found that our training protocols improve the antioxidant defense with particular effectiveness of ET (unpublished preliminary data). We are therefore conducting an ongoing specific study to improve the mechanistic knowledge of the BURST effect.

This result let us speculate that the ragged red fibers, resulting from mitochondrial oxidative damage and leading to weakening of muscle strength (Palmio and Udd, 2014), could be rescued and could be linked to better management of strength.

Even if in absence of fiber hypertrophy, a specific ragged red fiber rescue or other morpho-functional adaptations could be therefore positively linked to strength gain in BURST. We didn’t find any significant difference in the anthropometric characteristics from *t*0 to *t*1 in line with the results of Binns et al. (2017), who reported no significant difference in Lean body Mass after 20 weeks (2 sessions/w) of High Velocity Resistance Training in healthy old subjects (77 ± 6.4 y), both in males and in females. James et al. (2016) also reported no differences in healthy old males (55 to 80 years) on BMI, waist girth and hip girth after 6 and 12 months on intervention, based on an active lifestyle (30 min walking 3 times/w) or RT protocol (3 sessions/w). Thus, on healthy old males, our physical interventions were unable to improve significantly anthropometric characteristic after medium-term protocols.

## Conclusion

We extended the field of cross-transfer demonstrating for the first time the BURST in healthy elderly following training protocols dedicated to the lower limbs. In particular, the ET was the most effective protocol on HS gain.

We provided new insights for further researches: perspectively, we should investigate the underlying physiological mechanisms of BURST related to different training protocols on elderly. We suggest to define the role of “exerkines” and “exersomes” on this systemic cross-transfer.

The BURST effect in elderly should be considered in adapted physical activity, in the personalization of care and in rehabilitation.

In our opinion, the BURST is of high impact for the clinical implication in sarcopenic or atrophic muscle and, on the same time, it is very intriguing for scientific investigation.

## Author Contributions

TP designed the project, achieved the approval by Ethical Committee, received the funding, analyzed and discussed the data, and wrote the manuscript. DB analyzed and discussed the data, and wrote the manuscript. EK discussed the data, and revised the manuscript. VV recruited the participants, organized and supervised the training, and discussed the data. All authors approved the manuscript and agreed that the accuracy or integrity of any part of the work are appropriately investigated and resolved.

## Conflict of Interest Statement

The authors declare that the research was conducted in the absence of any commercial or financial relationships that could be construed as a potential conflict of interest.

## Abbreviations

1RM: 1 Repetition Maximum
BMI: Body Mass Index
BURST: Bottom-Up Rise Strength Transfer
ET: Endurance Training
FM: Fat Mass
HS: Handgrip Strength
MVC: Maximal Voluntary Contraction
RT: Resistance Training
